# Herpes Simplex Keratitis Following Corneal Crosslinking for Keratoconus: A One-Year Case Series Follow-Up

**DOI:** 10.3390/diagnostics14202267

**Published:** 2024-10-11

**Authors:** Freja Bagatin, Ivana Radman, Karla Ranđelović, Ivanka Petric Vicković, Valentina Lacmanović Lončar, Renata Iveković, Zoran Vatavuk

**Affiliations:** Department of Ophthalmology, Sestre Milosrdnice University Hospital Center, 10000 Zagreb, Croatia; frejabarisic@yahoo.com (F.B.); ivanaradman23@gmail.com (I.R.); karla.randelovic@gmail.com (K.R.); vlacmanovic@hotmail.com (V.L.L.); renata.ivekovic@kbcscm.hr (R.I.); zo.vatavuk@gmail.com (Z.V.)

**Keywords:** keratoconus, herpes simplex, keratitis, corneal cross linking

## Abstract

Corneal crosslinking (CXL) is a medical procedure used to treat keratoconus. CXL works by strengthening the collagen fibers of the cornea through the application of riboflavin (vitamin B2) and ultraviolet (UV) light, which helps to stabilize the cornea and prevent further deterioration. There is a recognized risk that CXL can trigger the reactivation of dormant herpes simplex virus (HSV), leading to herpetic keratitis even in patients with no history of herpetic disease. We examined the medical history of 52 patients who underwent CXL procedures due to previously diagnosed keratoconus. We reviewed the patient’s medical histories to assess whether there was a herpes labialis infection and/or herpetic keratitis. Altogether, 52 eyes (from 52 patients) were analyzed. Of those, four (7.69%) patients were diagnosed with epithelial herpetic keratitis on the 5–8th day after surgery. All four patients had a history of herpes labialis and no prior HSV keratitis infection. Two patients developed herpetic keratitis despite prophylactic therapy with acyclovir 5 days before surgery. A positive history of HSV lip infection before CXL was present in 18/52 (34.62%). During a one-year follow-up period, no patient experienced a recurrence. Close follow-up is crucial for diagnosing herpetic keratitis after corneal crosslinking. The use of prophylactic antiviral therapy in patients who are asymptomatic and have a history of recurrent herpes labialis does not guarantee the prevention of infection.

## 1. Introduction

Keratoconus is a disorder marked by progressive corneal thinning and steepening, which can lead to significant visual impairment due to high astigmatism, corneal scarring, or even corneal perforation [1,2,3].

Once detected, the primary goal in managing keratoconus is to slow or stop the advancement of the condition. Corneal collagen cross-linking (CXL) with riboflavin and ultraviolet A (UV-A) is currently the only treatment capable of halting or delaying the progression of keratoconus [4,5,6]. This technique utilizes the combined action of riboflavin, a photosensitizer, and UV-A rays to induce the formation of strong chemical bonds between collagen fibrils in the cornea, making it stiffer and less susceptible to ectatic changes [7,8]. 

The disruption of the corneal nerve plexus is a known complication during various ocular surgeries, including penetrating keratoplasty (PK), lamellar keratoplasty, CXL, cataract surgery, as well as photorefractive and phototherapeutic procedures. This trauma, combined with the modulation of the ocular immune response due to postoperative steroid use, can lead to the reactivation of herpes simplex keratitis (HSK).

While CXL is generally a safe and effective procedure, there are few reported complications, including infection, stromal haze, scarring, and endothelial toxicity [3,9,10,11]. Secondary HSK is a rare complication that can occur even in cases with no history of the disease [10,12,13,14,15].

Here, we describe a case series of patients who developed HSK following CXL.

## 2. Case Descriptions

We followed 52 eyes that underwent CXL surgery for progressive keratoconus at the Department of Ophthalmology, Clinical Hospital Center Sestre Milosrdnice in Zagreb, Croatia, from January 2022 to July 2023. 

None of the patients had a history of herpetic eye disease, ocular trauma, previous eye surgery, or other ocular or systemic diseases. Slit lamp examinations showed no signs of stromal scarring or clouding from past viral keratitis. Tests for dry eye disease (tear production, tear breakup time, corneal and conjunctival fluorescein staining) were negative. We analyzed medical histories and recorded patients who had herpes labialis before CXL as well as those that developed HSK after CXL. 

All patients underwent surgery on the eye with more advanced keratoconus. CLX surgeries were performed in a sterile operating room using the Dresden protocol [7]. Patients were informed of the benefits and risks and provided written consent before surgery. The surgical eye was anesthetized with topical 0.5% tetracaine eye drops, and 1% pilocarpine was used to constrict the pupil. Under sterile conditions, the surgeon mechanically removed the corneal epithelium at the central 8.0 mm area of the corneal surface using a blunt hockey knife or a 20% ethanol solution (applied for 20 s), after which the corneal epithelium was debrided with a spatula. Riboflavin 0.1% with hydroxypropyl methylcellulose 1.1% (MedioCROSS M^®^, MedioCROSS, Medio-Haus-Medizinprodukte GmbH Kiel, Kiel, Germany) was instilled every 2 min for 15 min before the procedure. The cornea was irradiated with UVA light at an irradiance of 3 mW/cm^2^ for 30 min (CSO Vega). During irradiation, riboflavin was continuously instilled at every 5-minute interval. This resulted in a total fluence (energy/surface area) of 5.4 J/cm^2^ [15]. After the procedure, a corneal bandage contact lens (Air Optix Night&Day^®^; Alcon, Minato City, Tokyo) was applied to the operated eye for 4–5 days. All patients were discharged the following day with ofloxacin eye drops 5 times a day, preservative-free artificial tears 5 times a day, and tobramycin-dexamethasone eye drops two times a day and asked to a follow-up after 5 days. Afterwards, CL was removed, and patients were instructed to continue with tobramycin–dexamethasone with gradual tapering.

Follow-up was on the first postoperative day, after 5 days, 1 month, 3 months, 6 months, and 1 year after CXL. At each follow-up examination, we recorded the best-corrected visual acuity, slit lamp examination, performed a Pentacam scan (Oculus Optikgeraete GmbH; Wetzlar, Germany) and anterior segment OCT (RTVue, Optovue, Fremont, CA, USA). Three months after CXL, we prescribed contact lenses.

Patients who developed HSK were monitored more frequently until the keratitis resolved and then every 3 months until one year after CXL.

All patients provided written informed consent for the publication of the case details and images. The study was performed according to the Declaration of Helsinki. 

Altogether, 52 eyes were analyzed. Of those, 40 were men (76.92%) and 12 women (23.08%) at a mean age of 32.5 years (18–47 years). 

A positive history of HSV infection of the lip before CXL was present in 18/52 (34.62%). After CXL, 4/52 (7.69%) patients, three men and one woman, aged 20–35 years, developed HSK, all of them with previous oral HSV infection. 

After the first two cases (patients 1 and 2) with HSV infection, we decided to introduce prophylactic therapy with acyclovir 5 days before surgery for the rest of the patients who have had herpes labialis (16/18, 88.89%). Despite this, 2/16 (12.5%) developed HSK after CXL (Table 1).

Table 2 summarizes basic information, clinical history, and pre-surgery eye examination results from four cases with an HSK complication. 

All four patients were diagnosed with epithelial herpetic keratitis 5–8th day (mean 6.5 days) after surgery based on clinical signs that included typical dendritic epithelial keratitis with terminal bulbs on slit-lamp. Details on clinical presentation, symptoms, and treatment are shown in Table 3. We were not able to perform a polymerase chain reaction (PCR) test to confirm the diagnosis of herpes infection, so the diagnosis was based on the clinical presentation of the lesions, prolonged epithelial healing time, reduced corneal sensation, and quick response to antiviral therapy. Patients were diagnosed on regular follow-up exams. The main symptoms were blurry vision, conjunctival injection, and tearing. Only patient 1 presented with eye pain. 

**Figure 1:**
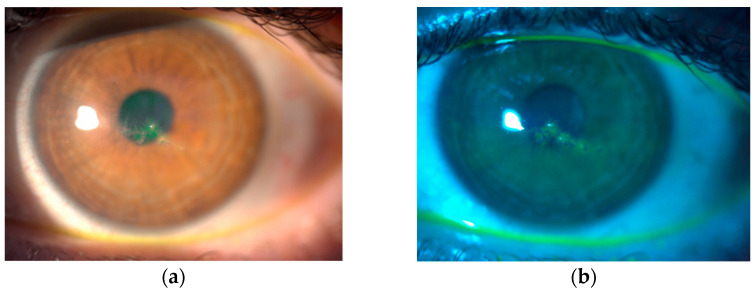
The initial slit lamp examination of the patient 1. (**a**) Corneal epithelial dendrite from herpes simplex keratitis on 5th postoperative day, before treatment; (**b**) Herpes simplex keratitis seen with fluorescein staining and a cobalt blue light.

**Figure 2:**
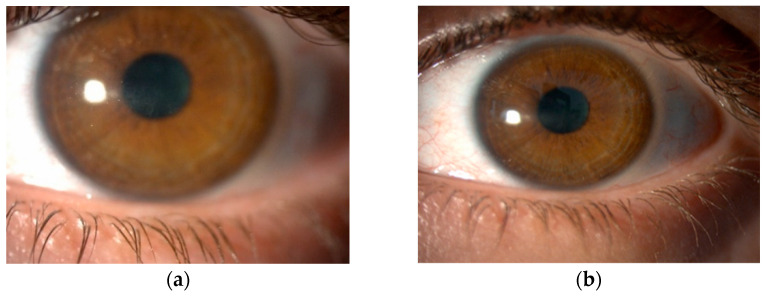
Follow-up. (**a**) Slit lamp image of the same patient 2 months after CXL, where we can see that the dendritic ulcer has completely healed. (**b**) Slit lamp image of the same patient 1 year after CXL.

After HSK diagnosis, the topical treatment was modified; topical acyclovir 3% was administered five times daily, and corticosteroid was discontinued. After complete epithelial healing, corticosteroids were continued in tapering mode. Oral acyclovir was started at a dose of 400 mg five times daily until the ulcer healed completely, followed by a reduced dosage of two times daily for a further 2 weeks. In addition, preservative-free artificial tears were used four times daily to promote reepithelization. Corneal healing time was 5–105 days (mean 55 days). Patients 1 and 3 had a dendritic ulcer with terminal bulbs and mild transient corneal opacification (Figure 1), while patient 2 had two dendritic ulcers. Patient 4 had the most severe clinical presentation, a geographic ulcer with stromal involvement. None of the patients showed signs of inflammation in the anterior chamber. All patients responded positively to the therapy. Patient 4 had a prolonged disease course lasting over 3 months with residual macula on the cornea, whereas the remaining three patients recovered within 3 weeks with no further corneal involvement (Figure 2a). During a one-year follow-up period, no patient experienced a recurrence of herpetic keratitis (Figure 2b). 

The comparison of our study results with other studies describing recurrence of HSK after CXL is presented in Table 4.

## 3. Discussion

Herpetic keratitis is an eye infection caused by the HSV, typically HSV-1. It affects the cornea and can lead to inflammation, scarring, and vision loss if not properly managed. HSV can remain dormant in the body and reactivate, potentially causing recurrent infections. Following the initial infection with HSV, individuals harbor the virus indefinitely [21]. Transmission usually happens through direct contact with infected secretions (i.e., saliva or tears) or lesions containing viral particles [22]. Although often asymptomatic, the initial infection can occasionally present as a nonspecific upper respiratory tract infection. HSV is commonly categorized into two types: HSV-1 and HSV-2 [23]. HSV-1 typically affects mucocutaneous areas supplied by the trigeminal nerve, such as regions around the mouth, face, and eyes. The virus spreads from infected epithelial cells to nearby sensory nerve endings and travels along the nerve axon to the trigeminal ganglion, where it establishes latency in the neuron’s nucleus. Research on non-human primates has shown that HSV-1 remains latent in the ciliary ganglion and cornea [24]. HSV infections, which can affect the corneal epithelium, stroma, or endothelium, often recur and may lead to permanent vision impairment. This can occur due to stromal scarring or endothelial dysfunction, especially in patients who do not respond well to antiviral therapy. HSK has been recognized as a potential complication following various ocular surgeries, including cataract surgery, vitrectomy, laser-assisted in situ keratomileusis (LASIK), penetrating keratoplasty (PK), and descemet membrane endothelial keratoplasty (DMEK). HSK may arise from either reactivation of latent herpes simplex virus (HSV) or a new primary infection.

The percentage of herpes simplex keratitis (HSK) reactivation after various ocular surgeries can vary based on the type of surgery and patient characteristics. The incidence of HSK after corneal transplantation can range from 0.73% to 6% [25,26,27]. Reports suggest an incidence of 1% to 2% of HSK in post-cataract surgery [28] and lower incidence after photorefractive keratectomy (PRK) and LASIK (0.14% and less than 0.1%, respectively) [29,30].

Factors triggering HSV reactivation in the cornea include ultraviolet exposure, ocular surgery, topical steroids, topical latanoprost, emotional stress, illness, and immunosuppression [31,32]. CXL may involve epithelial and stromal trauma, damage to the subepithelial nerve plexus, UV-A irradiation, and the use of topical steroid drugs, all of which can trigger reactivation of latent HSV infections even in patients with no previous history [33]. Therefore, it is contraindicated to perform the CXL procedure in patients with a previous episode of HSK, as this may cause reactivation and corneal melting, as described in some cases [3,10,34]. 

Crosslinking has been found to be well tolerated and effective in reducing the progression of ectatic corneal diseases such as keratoconus [3]. As shown in a recent study, it stabilizes both the tomographic and biomechanical properties of the cornea for up to four years after the procedure [35]. Nevertheless, CXL surgery may, in rare cases, trigger the reactivation of the HSV, potentially leading to vision-threatening complications if not managed promptly and effectively. Keratitis following corneal cross-linking (CXL) has been linked to several factors, including the postoperative use of soft bandage contact lenses, topical corticosteroids, and the presence of epithelial defects, as well as potential adverse effects of UV light on corneal immune mechanisms and wound healing processes. 

There are a few main differences between CXL and other ocular surgeries:

1. CXL involves the application of riboflavin (vitamin B2) and ultraviolet light to strengthen the corneal tissue. This process disrupts the corneal nerve plexus and can decrease corneal sensitivity, making it easier for latent HSV to reactivate. The herpetic eye disease study (HEDS) trial also evaluated psychological stress, infection, and exposure to sunlight as potential ocular HSV triggers [31]. Procedures like cataract surgery or glaucoma surgery typically do not involve the same level of corneal manipulation or disruption of the nerve plexus, which may result in a lower risk of HSK reactivation.
2. According to our study, the incidence of HSK following CXL can be relatively high due to surgical trauma and changes in the corneal environment. While HSK can occur after other ocular surgeries, the rates are often lower and more variable, depending on the specific procedure and the patient’s history of HSV.
3. Reactivation of HSK may occur more promptly following CXL due to immediate postoperative changes in the cornea and immune response. Reactivation after other surgeries may happen but often occurs later in the postoperative course, depending on the type of surgery.
4. The healing process post-CXL can be prolonged, and the compromised corneal epithelium can increase vulnerability to viral reactivation during this time. Recovery after other surgeries like cataract removal is typically quicker, with less risk of prolonged epithelial compromise, potentially leading to a lower risk of HSK.

Several cases of HSK following corneal CXL have been reported, but this is not a commonly described complication after CXL surgery [12,14,17,18,19] (Table 4). Serraro et al. reviewed adverse event rates from 25 publications covering 9607 eyes, with 9006 undergoing epi-off CXL procedures. For epi-off procedures, the rate of HSK was 0.18% (4/2182) [20]. Wang showed a case series of 4 patients with newly-onset HSK out of 300 patients receiving corneal CXL surgery, which shows an incidence of approximately 1.33% (4/300) [18]. A recent study showed a retrospective analysis of the medical history of 543 patients who underwent CXL surgery. There were nine cases of HSK, indicating an incidence of 1.65% [16]. In the present study, there were 4 out of 52 (7.69%) cases of HSK, which is significantly higher compared to previous studies. The fact that we found four cases in a one-year cohort of only 52 patients prompted us to present these results.

The higher rate of HSK in our study could be due to the small sample size. Other possible explanations for the higher HSK rate could be a higher number of individuals with a genetic predisposition that can increase susceptibility to the HSV or who have previously experienced HSV episodes, such as herpes labialis [36]. Reduced immune response or environmental factors can also facilitate the reactivation of the HSV. In a large retrospective case-control study in California, individuals with severe atopic disease had between 2.0 and 4.8-fold greater odds of developing ocular HSV than those without atopy [37]. Additionally, patients with atopy have also been noted to have unusually severe HSV keratitis [38]. 

In our cases, patients did not have a previous positive medical history of HSK. However, primary HSV ocular infection is often asymptomatic or mild and easily overlooked, so this can be a major challenge when taking patients’ clinical histories. In other studies, patients also did not have a previous positive medical history of HSK, and history of herpes labialis was rarely recorded [12,17,18,19]. Only in one study a patient had previous herpes labialis and was advised to take acyclovir 400 mg orally five times a day 3 days prior to surgery as prophylaxis. However, despite this, he developed keratitis, and the ulcer healed slowly regardless of oral therapy [16]. Out of the 52 patients in this study, 18 had a previous herpes labialis before CXL, including all four patients who developed HSK postoperatively. A high incidence of labial herpes in our group may be the reason for the observed higher prevalence of HSK. 

Individuals who have previously experienced episodes of HSV, such as herpes labialis, are known to be at a greater risk of developing HSK [36]. Some authors have suggested prophylactic antiviral therapy during the perioperative period of ocular surgeries to reduce the risk of HSV reactivation [39,40]. Laser corneal refractive surgery, keratoplasty, cataract surgery, and CXL alter the sub-basal nerve plexus of the cornea. The surgical trauma and the modulation of the ocular immune response by postoperative steroids can potentially induce the reactivation of HSK. However, there is no consensus regarding the suitable candidates, drug regimen, or timing for initiating antiviral therapy before ocular surgery in general [41]. Since our patients 1 and 2 developed keratitis, we decided to administer prophylactic treatment to all patients with herpes labialis (oral acyclovir 400 mg two times daily) 5 days prior to treatment, until the cornea had healed completely and the contact lens was removed. Patients 3 and 4 received prophylactic treatment but still developed HSK, which in patient 4 lasted longer and was more severe. There remains a question of whether corneal CXL can lead to recurrent episodes of HSK in these patients in the future. In our case group, after a one-year follow-up, there were no recurrent episodes of HSK. Future larger studies should concentrate on the long-term prognosis of patients who develop new-onset HSK after CXL surgery and gather evidence regarding the use of prophylactic antiviral therapy for these individuals. Even though prophylactic systemic antiviral treatment in patients with a history of HSK might decrease the possibility of recurrence, there is still a substantial risk, as 12.5% of patients in our series developed HSK despite prophylaxis. 

This study on HSK after CXL surgery is important for several reasons. First, this is the first case series of HSK following corneal CXL surgery with a one-year follow-up. Secondly, it provides insights into the incidence and risk factors for HSK following CXL, helping to clarify potential complications that can arise from this increasingly common procedure. The findings can help shape postoperative care protocols, including the use of antiviral prophylaxis, and guide clinicians in managing patients with a history of HSV. This research adds to the existing knowledge by focusing specifically on the intersection of HSK and cross-linking, a topic that is not comprehensively covered in previous studies. The study can also highlight areas for future research, such as the long-term effects of CXL on corneal immunity and healing and the development of more effective strategies to prevent viral reactivation.

There are a few limitations to our study. The surgeries were performed by three different surgeons, one performing a mechanical removal of the corneal epithelium using a blunt hockey knife while two other surgeons used a 20% ethanol solution, after which the corneal epithelium was debrided with a spatula. There is a lack of consistency that may be introduced by a single surgeon; however, it may be informative to see whether different methods influence the outcome. In our case series, patients 3 and 4 had alcohol-assisted delamination (ALD), and patients 1 and 2 had mechanical removing of the corneal epithelium. Due to its effectiveness in achieving a smooth cleavage plane between the epithelium and Bowman’s layer, ALD of the corneal epithelium has been widely utilized for various diagnostic and therapeutic purposes, occasionally yielding both outcomes [42]. In a previously published retrospective case series, which included patients undergoing epithelium-off CXL for keratoconus using ALD for epithelial removal in 36 eyes, only one case of sterile corneal infiltrates (2.78%) was reported. No other complications were observed [43]. Whether ALD or manual epithelial removal can predispose to HSK cannot yet be confirmed.

Additionally, we did not have the facility for polymerase chain reaction (PCR) for confirmation of the diagnosis of HSV infection. The diagnosis was made based on very straightforward clinical signs. The PCR test can detect viral DNA and quantify the number of viral copies, distinguishing between viral shedding and replication. Variable reports of the sensitivity of PCR testing exist in different studies, from 34% [44] where the HSV PCR test had an overall low positive rate to a very high rate (88–100%) [45,46]. In all studies presented, regardless of the PCR result, most patients improved with the initial antiviral therapy, especially for epithelial HSK. Thus, a clinical diagnosis of HSK might be adequate to guide treatment [44].

## 4. Conclusions

To our knowledge, this study represents the first case series of HSK following corneal CXL surgery, with a one-year follow-up.

Reactivation of HSV after corneal surgery can occur, but the frequency can vary depending on several factors, such as the patient’s medical history, the type of surgery performed, and whether prophylactic antiviral therapy was used. It is essential for clinicians to monitor patients closely for any signs of HSV reactivation post-surgery and to consider prophylactic measures to mitigate the risk. Physicians should be aware of the risks of ocular HSV reactivation with UVA light and inform patients of this risk before the procedure. It is also important to collect data on previous HSV infections such as herpes labialis, as a high rate of patients with previous herpes labialis infection in our patient group may be the reason for a higher number of HSK cases compared to other studies.

While episodes can resolve on their own, early treatment of the infection is crucial to minimize viral replication, shorten the duration of the disease, and maintain latency, thereby preventing further complications. The prophylactic use of antiviral therapy in patients with a positive history of HSV infection may be beneficial, but prevention of HSK is not always possible.

## Figures and Tables

**Table 1:** Data on HSV infection and implemented prophylaxis.

	Male/All (%)	Female/All (%)	Total/All (%)
Previous oral HSV infection	10/52 (19.23%)	8/52 (15.38%)	18/52 (34.62%)
Corneal HSV infection after CXL	3/52 (5.77%)	1/52 (1.92%)	4/52 (7.69%)
HSV oral prophylaxis 5 days before surgery	14/18 (77.78%)	2/18 (11.11%)	16/18 (88.89%)
Corneal HSV infection after CXL with oral prophylactic treatment	1/14 (7.14%)	1/2 (50%)	2/16 (12.5%)

HSV—herpes simplex virus; CXL—corneal crosslinking.

**Table 2:** Demographic and pre-operative clinical data on patients that developed HSK after corneal crosslinking surgery.

	Patient 1	Patient 2	Patient 3	Patient 4
Gender	M	M	F	M
Age	20	34	35	35
Surgical eye	right	left	right	right
KC stage	I–II	III	II–III	II
History of herpetic eye disease	NO	NO	NO	NO
History of labial herpes	YES	YES	YES	YES
Preoperative acyclovir prophylaxis 400 mg b.i.d.	NO	NO	YES	YES
Atopy	YES	NO	NO	NO
Previous intraocular or corneal surgery	NO	NO	NO	NO
History of RGP CL use	NO	NO	NO	YES
BCVA before CXL surgery	0.2	0.6 pinhole 0.7	0.2 pinhole 0.6	0.9
Corneal topography (pentacam)	K1 48.7 K2 56.4 K max 68.7 PV 422 TL 413	K1 44.5 K2 49.9 K max 57.8 PV 495 TL 491	K1 46.4 K2 50.9 K max 54.6 PV 455 TL 445	K1 42.3 K2 46.5 K max 54.3 PV 465 TL 454

KC—keratoconus; b.i.d.—twice a day; RGP CL—rigid gas-permeable contact lens; BCVA—best corrected visual acuity according to Snellen; CXL—corneal crosslinking; K1—flat keratometry; K2—steep keratometry; K max—maximum keratometry reading; PV—corneal thickness measurement taken at the vertex of the cornea; TL—thinnest location of the cornea.

**Table 3:** Clinical manifestations of herpetic keratitis after corneal crosslinking surgery.

	Patient 1	Patient 2	Patient 3	Patient 4
Time of onset	5th postop. day	8th postop. day	6th postop. day	8th postop. day
Patients’ symptoms	Eye pain, blurry vision, conjunctival injection, tearing	No eye pain, blurry vision, conjunctival injection, tearing	No eye pain, conjunctival injection, tearing	No eye pain, blurry vision, conjunctival injection, tearing
Clinical signs of HSV infection	Dendritic epithelial ulcer, mild transient corneal opacification	Two Dendritic epithelial ulcers	Dendritic epithelial ulcer	Geographic epithelial ulcer, stromal corneal involvement
Treatment	Oral acyclovir 400 mg q.d. Topical acyclovir 3% q.d. until the cornea ulcer healed and then t.i.d. for a week, Topical Ofloxacin q.d. Topical carboxymethylcellulose q.d. Topical dexamethasone q.i.d.	Oral acyclovir 400 mg q.d. Topical acyclovir 3% q.d. until the cornea ulcer healed and then t.i.d. for a week Topical Ofloxacin q.d. Topical carboxymethylcellulose q.d. Topical dexamethasone q.i.d.	Oral acyclovir 400 mg q.d. Topical acyclovir 3% q.d. until the cornea ulcer healed and then t.i.d. for a week Topical Ofloxacin q.d. Topical carboxymethylcellulose q.d. Topical dexamethasone q.i.d.	Oral acyclovir 400 mg q.d. Topical acyclovir 3% q.d. until the cornea ulcer healed and then t.i.d. for a week Topical Ofloxacin q.d. Topical carboxymethylcellulose q.d. Topical dexamethasone q.i.d.
Time to complete ulcer healing	10 days	5 days	20 days	105 days
Recurrence of HSK during a one-year follow-up	NO	NO	NO	NO
Clinical signs at last visit	NO	NO	NO	Corneal macula
BCVA at last visit (after one year)	1.0	0.7	RK2 CL 0.8	RK2 CL 0.6–0.7

HSV—herpes simplex virus; q.d.—five times a day; t.i.d.—three times a day; q.i.d.—four times a day; HSK—herpes simplex keratitis; BCVA—best corrected visual acuity according to Snellen; RK2—Rose K2; CL—contact lens.

**Table 4:** Post-corneal crosslinking herpes simplex keratitis in present study and published reports.

Study	Type of Study	Number of Eyes/Cases	Previous HSV Labialis/Keratitis	Time of HSK on-Set after Surgery	Clinical Presentation	Follow-Up Period (Months)
Wroblewska-Czajka et al., 2021 [16]	Retrospective study	9/543	Yes/No	2–6 days	herpes keratitis, iritis	49.3 months
Kymionis et al., 2007 [17]	Case report	1	No/No	5 days	geographic epithelial keratitis and iritis	2 months
Wang et al., 2022 [18]	Case series	4/300	No/No	3 days–1 month	corneal opacity and stromal edema	2 weeks 2 months 1 week
Yuksel et al., 2011 [19]	Case report	1	No/No	4 days	dendritic ulcer	1 month
Awad Al-Qarni, 2015 [12]	Case report	2	No/No	6 and 9 days	dendritic ulcer	4 months
Sitaula et al., 2019 [14]	Case report	2	No/No	7 days	bilateral herpes keratitis	1 month
Serrao et al., 2021 [20]	Literature review	4/2182	No/No	-	herpes keratitis	12–36 months
Our Study (Bagatin et al., 2024)	Case study	4/52	Yes/No	5, 6, and 8 days	dendritic epithelial ulcer	12 months

## Data Availability

The raw data supporting the conclusions of this article will be made available by the authors on request.
